# Excessive neutrophil extracellular trap formation induced by *Porphyromonas gingivalis* lipopolysaccharide exacerbates inflammatory responses in high glucose microenvironment

**DOI:** 10.3389/fcimb.2023.1108228

**Published:** 2023-01-20

**Authors:** Yue Tong, Yue Xin, Lanqing Fu, Jia Shi, Ying Sun

**Affiliations:** ^1^Department of Periodontology, The Affiliated Stomatological Hospital of Nanjing Medical University, Nanjing, China; ^2^Jiangsu Province Key Laboratory of Oral Diseases, Nanjing, China; ^3^Jiangsu Province Engineering Research Center of Stomatological Translational Medicine, Nanjing, China

**Keywords:** *Porphyromonas gingivalis*, lipopolysaccharide, neutrophil extracellular traps, reactive oxygen species, inflammatory responses

## Abstract

**Introduction:**

Neutrophil extracellular trap (NET) is a novel defense strategy of neutrophils and found to be induced by Porphyromonas gingivalis (P. gingivalis) lipopolysaccharide (LPS) or high glucose. The aim of this study was to investigate the roles and mechanisms of NET formation in high glucose inflammatory microenvironment.

**Methods:**

NETs induced by 1 μg/ml P. gingivalis LPS and/or 25 mM glucose were visualized using a fluorescence microscopy and the levels of extracellular DNA were determined by a microplate reader. The bactericidal efficiency of NETs was assessed by quantifying the survival P. gingivalis in neutrophils. The levels of NLRP3 and IL-1β in THP-1 derived-macrophages, and the expressions of p-PKC βII, p-MEK1/2, p-ERK1/2, ORAI1 and ORAI2 in neutrophils were detected by Western blot. Moreover, levels of intracellular Ca2+ and reactive oxygen species (ROS) in neutrophils were explored by flow cytometry.

**Results:**

P. gingivalis LPS enhanced the formation of NETs and increased the levels of extracellular DNA in high glucose microenvironment (p < 0.05). Compared with normal glucose inflammatory microenvironment, quantities of extra- and intracellular viable P. gingivalis in neutrophils exposed to NETs induced in high glucose inflammatory one were increased (p < 0.05) and the expressions of NLRP3 and IL-1β were dramatically increased in macrophages co-cultured with NETs from high glucose inflammatory microenvironment (p < 0.05). In addition, levels of ROS, intracellular Ca^2+^, p-PKC βII, p-MEK1/2, p-ERK1/2, ORAI1 and ORAI2 were increased in neutrophils stimulated with both high glucose and P. gingivalis LPS compared with the single stimulus groups (p < 0.05).

**Discussion:**

In high glucose inflammatory microenvironment, formation of NETs was enhanced via oxidative stress, which failed to reverse the decreased bactericidal capacity in high glucose microenvironment, and instead aggravated the subsequent inflammatory responses.

## Introduction

Periodontitis is a chronic inflammatory disease caused by a variety of periodontal pathogens and eventually leads to alveolar bone resorption and tooth loss (Hajishengallis, 2022). *Porphyromonas gingivalis* (*P. gingivalis*), a member of the red complex, is one of the most important periodontal pathogens (Chigasaki et al., 2021). Lipopolysaccharide (LPS), a cell wall component of *P. gingivalis*, triggers immune and inflammatory responses in the host due to its potential toxicity and antigenicity (Xu et al., 2020).

As two major global health threats, diabetes and periodontitis have received a lot of attention in recent years. Growing evidences suggest a bidirectional relationship between these two diseases (Salhi and Reners, 2022). Periodontitis is the sixth most common complication of diabetes. Epidemiological evidences indicate that people with diabetes are at high risk for severe periodontitis (Genco and Borgnakke, 2020), while there is an increased incidence of diabetes in periodontitis patients (Genco et al., 2020). On the one hand, serve periodontal infection impairs insulin sensitivity and worsens glycemic control. On the other hand, diabetes exacerbates oxidative stress and inflammation, which contributes to serious immune damages in periodontal tissues (Polak et al., 2020). However, the exact mechanisms by which the two diseases interact are not fully understood.

As the most abundant circulating leukocytes in peripheral blood, neutrophils play an important role in innate immune responses (Liew and Kubes, 2019). They are recruited to the site of infection, where they kill invading microorganisms by phagocytosis, degranulation, release of reactive oxygen species (ROS) and cytokine production (Burn et al., 2021). In addition, cross-talk between neutrophils and other immune cells, such as macrophages, natural killer, dendritic, B and T lymphocytes, are disclosed by accumulating evidences (Tsai et al., 2021; Herrero-Cervera et al., 2022). Normally, there are increased numbers of neutrophils with declinied biological functions, including impaired phagocytosis, adhesion and chemotaxis, delayed apoptosis and enhanced release of inflammatory mediators in diabetes patients, which leads to excessive inflammatory responses and immune destructions in periodontal tissues (Graves et al., 2020; Dowey et al., 2021; Giovenzana et al., 2022).

In 2004, a novel bactericidal strategy, neutrophil extracellular traps (NETs) were firstly found by Brinkmann (Brinkmann et al., 2004). NETs can be induced by various stimulations, such as PMA, LPS, IL-8 and so on, and are characterized as decondensed extracellular DNA structures decorated with a variety of antibacterial proteins, including histones, neutrophil elastase and myeloperoxidase (MPO) (Wang et al., 2021). Invading pathogens can be entrapped by NETs, and then be killed. However, excessive NET production may lead to tissue damages in the host and are related with a broad spectrum of autoimmune diseases and cancers (Ravindran et al., 2019).

Roles of NETs in the development of periodontitis are gradually uncovered. Abundance of NETs was detected in purulent crevicular exudate and gingival crevicular fluid (GCF) from patients with periodontitis (Magán-Fernández et al., 2020). NETs may be powerful protectors of periodontal tissues, involving in the capturing and killing periodontal pathogens, as well as resolving periodontal inflammation. However, exaggerated NETs may cause epithelial cytotoxicity, damage basal lamina and impair tissue regeneration (Vitkov et al., 2020). Peptidylarginine deiminase 4 (PAD4) and Ca^2+^ was crucial for *P. gingivalis* LPS-induced NET formation (Chen et al., 2022). Meanwhile, high glucose promotes the production of NETs, which is closely associated with the development of diabetes and its complications (Njeim et al., 2020). NADPH oxidase-derived ROS was also involved in NET formation in diabetic retinopathy (Wang et al., 2019). Excessive NET formations in diabetic patients may lead to extracellular matrix degradation and delayed wound healing (Sabbatini et al., 2021). However, it still remains obscure whether the effects of diabetes on the development of periodontitis can be achieved by modulating NET formation in high glucose inflammatory microenvironment, and what exact regulatory mechanisms are involved. In the present study, it was hypothesized that exposure to *P. gingivalis* LPS and high glucose might have synergistic effects on NET formation. The possible involvement of oxidative stress and intracellular Ca^2+^ in the regulation of NET production in neutrophils stimulated with *P. gingivalis* LPS combined with high glucose was also explored.

## Materials and methods

### Reagents

*P. gingivalis* ATCC 33277 LPS were purchased from InvivoGen (CA, USA). SYTOX Green was acquired from Life Technologies (CA, USA). 2′,7′-Dichlorofluorescein diacetate (DCFH-DA) was obtained from Sigma-Aldrich (MI, USA). Anti-MPO, ORAI1, ORAI2 and NLRP3 antibodies was supplied by Abcam (Cambridge, UK). Antibody against PCK βII was from Huabio (Hangzhou, China) and primary anti-bodies against ERK1/2, p-ERK1/2, MEK, p-MEK and p-PKC βII were gotten from Cell Signaling (MA, USA). Antibodies to IL-1β and β-actin were provided by Affinity (OH, USA).

### Cell culture

This study was approved by the Ethical Committee Department, The Affiliated Stomatological Hospital of Nanjing Medical University in accordance with the ethical guidelines of the Declaration of Helsinki and written informed consents were obtained from all recruits.

Neutrophils were isolated from peripheral venous blood collected from systemically and periodontally healthy donors by density gradient centrifugation (Silvestre-Roig et al., 2019). The purity and viability of isolated neutrophils were more than 95%, which were determined by Diff-Quik staining and flow cytometry, respectively. Then, the freshly isolated neutrophils were resuspended in RPMI 1640 medium (Gibco, USA) containing 5.5 mM (normal glucose, NG) or 25 mM (high glucose, HG) glucose for subsequent experiments.

THP−1 cells were obtained from Shanghai Institutes for Biological Sciences, Chinese Academy of Sciences (Shanghai, China) and incubated in RPMI 1640 medium with 100 ng/mL PMA (Sigma, Germany). After a 48 h-differentiation, nonattached cells were aspirated and the remaining adherent macrophages were cultured in fresh RPMI 1640 medium for additional 24 h.

### NET detection

Neutrophils (5 x 10^5^ cells/well) were cultured in 12-well plates and divided into four groups. Group 1 and 3 were incubated in RPMI 1640 medium containing 5.5 mM glucose. Group 2 and 4 were incubated in medium containing 25 mM glucose. Both Group 3 and 4 were stimulated with 1 μg/ml *P. gingivalis* LPS for 3 h.

To visualize NETs, neutrophils (5 x 10^5^ cells/well) were seeded on poly-L-lysine-coated slides in 12-well plates. After a 3 h-stimulation, cells were fixed with 4% paraformaldehyde, permeabilized with 0.2% Triton X-100, blocked with 2% bovine serum albumin and then incubated with antibody against MPO (1:250) at 4°C overnight. Subsequently, neutrophils were incubated with Alexa Fluor 488–conjugated anti-rabbit IgG (H + L) (Beyotime, China) for 3 h and stained with DAPI (Beyotime, China) for 2 min. Finally, NETs were observed by a fluorescence microscope (Leica, Germany) and percentages of neutrophils forming NETs under 3 random fields of views were counted.

In addition, NET formation was quantified by SYTOX Green staining as described in previously study (Chen et al., 2022). Briefly, 5 μM SYTOX Green were added into the conditioned culture medium. After 30 min, the amounts of extracellular DNA in culture medium were detected by SpectraMax M2e microplate reader (Molecular Devices, Germany) using 504 nm excitation and 523 nm emission filters.

### Bacterial killing assay

*P. gingivalis* ATCC 33277 was kindly provided by Jiangsu Province Key Laboratory of Oral Diseases (Nanjing, China). It was cultured on brain heart infusion (BHI) agar plates containing 5% sheep blood anaerobically for 5-7 days and then enriched in BHI broth for an additional 24-72 h. Neutrophils (5 x 10^5^ cells/well) were cultured in 12-well plates and divided into five groups. Group 1-4 were stimulated as described before. Group 5 (blank control group) was blank medium containing 5.5 mM glucose without neutrophils. All groups were challenged with *P. gingivalis* at a MOI of 100:1 for 3 h. Cells were lysed with deionized water for 10 min to release intracellular bacteria and cultured on BHI agar plates anaerobically for 7-10 days. **The number of viable bacteria** relative to blank control group was calculated to evaluate the bactericidal efficiency of NETs in each group.

### NET isolation and co-culture with THP-1 cells

Neutrophils (1 x 10^6^ cells/well) were divided into four groups. Group 1 and 2 were stimulated with 1 μg/ml *P. gingivalis* LPS in RPMI 1640 medium containing 5.5 mM glucose for 3 h. Group 3 and 4 were stimulated with 1 μg/ml *P. gingivalis* LPS in medium containing 25 mM glucose for 3 h. Then, group 2 and 4 were incubated with 5 μg/ml DNase I (Sigma, USA) for 1 h to digest NETs. NET-containing supernatants were collected and mixed with an equal volume of fresh RPMI 1640 medium. THP−1-derived macrophages were cultured with the prepared conditioned medium for 12 h and then collected for subsequent western blot.

### Cytosolic Ca^2+^ measurement

Fluo-4AM (Beyotime, China) is a Ca^2+^ fluorescent dye that can penetrate cell membrane. Neutrophils were divided into 4 groups as described in 4.3 and incubated with 3 μM Fluo-4AM for 30 min. Fluorescence intensity of intracellular Ca^2+^ was measured by flow cytometry (FACSCalibur, BD, USA).

Two potent Ca^2+^ chelators, EGTA and BAPTA, can chelate free extracellular and intracellular Ca^2+^, respectively. To confirm the roles of cytosolic Ca^2+^ in NET formation, neutrophils were pretreated with 1 mM EGTA for 30 min or 5 μM BAPTA for 50 min in dark. Then, the levels of Ca^2+^ and extracellular DNA were measured as described previously.

### Detection of oxidative burst product

To measure the production of ROS, neutrophils(1 x 10^6^ cells/well)were grouped as described in 4.3 and incubated with 10 μΜ DCFH-DA, an oxidation-sensitive fluorescent probe, for 40 min. Then, cells were washed, resuspended in ice-cold PBS and measured immediately by FACSCalibur flow cytometer.

NADP^+^/NADPH ratio in neutrophils was detected using a NADP^+^/NADPH Assay Kit (Beyotime, China) based on WST-8 assay. Neutrophils were stimulated with 1 ug/ml *P. gingivalis* LPS in RPMI 1640 medium containing 5.5 mM or 25 mM glucose and lysed with NADP^+^/NADPH extraction buffer on ice. After a-12,000 g centrifugation for 10 min, the supernatant was collected and separated into two portions. One portion was heated at 60°C for 30 min to destroy NADP^+^ (only NADPH left), while the other was left on ice to detect both NADP^+^ and NADPH. Each of the two parts was individually mixed with the working solution for 10 min and then measured at 450 nm spectrophotometrically.

To further confirm the roles of ROS in NET production, neutrophils were pretreated with 10 μM Diphenylene iodonium (DPI), an inhibitor of NADPH oxidase, or 5.9 nM LY333531, an inhibitor of PKC βII, for 30 min, and then incubated with SYTOX Green dye to explore the levels of extracellular DNA.

### Western blotting

Neutrophils and THP-1 derived-macrophages were harvested in an ice-cold RIPA buffer (Beyotime, China) for protein extraction. After a-10% SDS-PAGE and electrotransfer, membranes were blocked with 5% non-fat milk, incubated with primary antibodies against MEK, p-MEK, ERK, p-ERK, PKC βII, p-PKC βII, ORAI1, ORAI2, NLRP3, IL-1β and β-actin at 4°C overnight. After an incubation with goat anti-rabbit or goat anti-mouse secondary antibody for 1 h, the proteins were detected with an ECL chemiluminescence kit (Beyotime, China) and semi−quantitively analysis using Image J software (National Institutes of Health, MD, USA) was then performed. All results were normalized to the levels of β-actin, and phosphorylated proteins were expressed as the relative gray value to non-phosphorylated proteins.

### Statistical analyses

All data are presented as mean ± SD. Statistical analysis were performed using one-way ANOVA and differences between groups were compared by LSD test. p value less than 0.05 was regarded as statistically significant.

## Results

### High glucose promoted the formation of NETs

**Figure 1:**
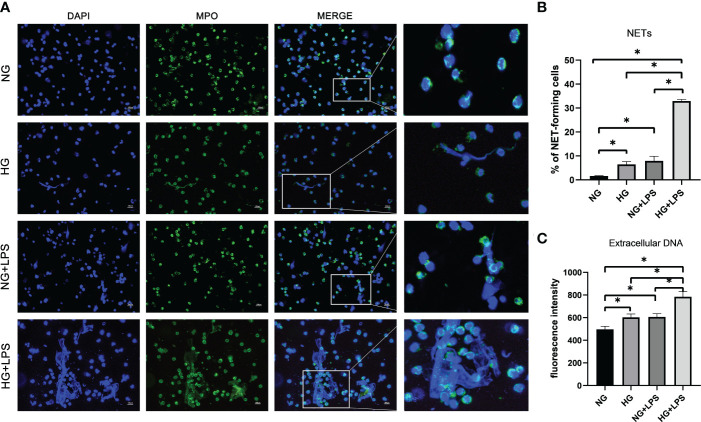
P *gingivalis* LPS exacerbated NET formations in a high glucose microenvironment. Neutrophils were stimulated with 1 μg/ml *P. gingivalis* LPS for 3 h in culture medium with 5.5 mM glucose or 25 mM glucose. **(A)** Cells were stained with an antibody to MPO (green) and DAPI (blue), and then visualized by an immunofluorescence microscopy. One representative result of three independent experiments is shown. Scale bar was 100 μm. **(B)** Percentage of NET-forming neutrophils to total cells counted was calculated. **(C)** Quantities of extracellular DNA were determined by Sytox Green assay. Data are presented as mean ± SD (n = 3 donors per group). *p < 0.05.

NET formation was firstly observed by immunofluorescence microscopy. Without *P. gingivalis* LPS stimulation, neutrophils in NG medium (5.5 mM) were founded to be round-like cells and there was almost no extracellular DNA network formation. Extracellular network-like DNA strands (blue) decorated with MPO (green) were observed in neutrophils stimulated with HG (25 mM) and/or *P. gingivalis* LPS, indicating the formation of classical NETs (**Figure 1A**). Moreover, there were more NET-positive cells in HG + *P. gingivalis* LPS group compared with the rest of the groups (p < 0.05, **Figure 1B**).

Quantification analysis of extracellular DNA was consistent with NET visualization. It was revealed that both HG and *P. gingivalis* LPS stimulation increased the levels of extracellular DNA compared with those in unstimulated group (p < 0.05, **Figure 1C**), and the level of extracellular DNA in HG + *P. gingivalis* LPS group was much higher than those with a single stimulus (p < 0.05, **Figure 1C**).

### Enhanced formation of NETs failed to reverse the decreased bactericidal ability of neutrophils in high glucose microenvironment

**Figure 2:**
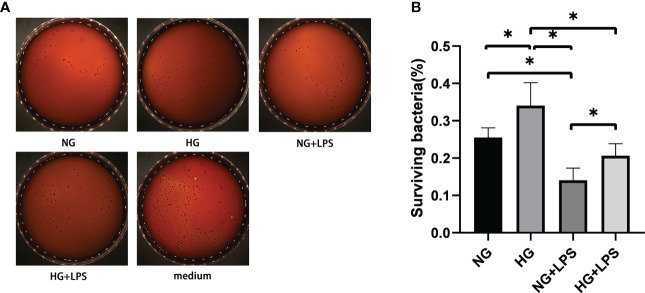
Enhanced formation of NETs was inconsistent with the decreased bactericidal ability of neutrophils in high glucose microenvironment. Neutrophils in culture medium with 5.5 mM or 25 mM glucose were treated with 1 μg/ml *P. gingivalis* LPS for 3 h. NG (5.5 mM) culture medium without neutrophils served as a negative control. Then, neutrophils in conditioned supernatants containing NETs were stimulated with *P. gingivalis* at a MOI of 100:1 for another 3 h. Cells were lyzed with ultrapure water and living *P. gingivalis* on agar plates were counted after 7-10 days. **(A)** Representative result of three independent experiments is shown. **(B)** The percentage of surviving bacteria was calculated by plate counting method. Data are presented as mean ± SD (n = 3 donors per group). *p < 0.05.

Previous studies have revealed the roles of NETs in enhancing the efficacy of neutrophils in killing periodontal pathogens (Chen et al., 2022). In HG culture medium, *P. gingivalis* LPS induced more NET formation, and then bactericidal activities of neutrophils exposed to these NETs were explored by bacterial viability assays (**Figure 2A**). The number of viable bacteria represents *P. gingivalis* not killed by neutrophils. The amount of viable of *P. gingivalis* was significantly decreased in NG + *P. gingivalis* LPS group compared with NG group (p < 0.05, **Figure 2B**). Despite the enhanced formation of NETs, the quantities of viable bacteria in HG group were significantly more than those in NG group (p < 0.05, **Figure 2B**). Furthermore, a similar increase in the amount of viable bacteria were also found in HG + *P. gingivalis* LPS group compared with NG + *P. gingivalis* LPS group (p < 0.05).

### *P. gingivalis* LPS and glucose-induced NETs contributed to the up-regulated expressions of Nod-like receptor family, pyrin domain containing 3 and IL-1β in macrophages

**Figure 3:**
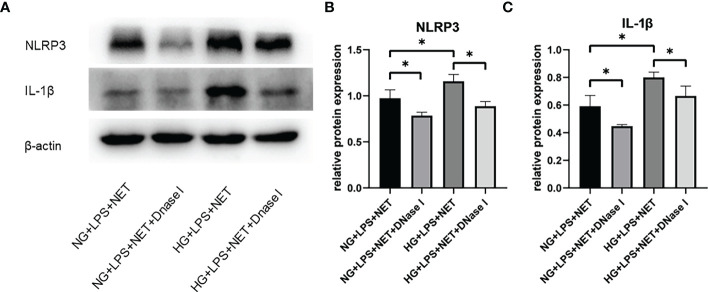
NETs induced in high glucose inflammatory microenvironment contributed to NLRP3 inflammasome activation and IL-1β release in macrophage. Neutrophils in 5.5 mM or 25 mM glucose culture medium were stimulated with 1 μg/ml *P. gingivalis* LPS and then incubated with 5 μg/ml DNase I for 1 h. THP-1-derived macrophages were incubated with supernatants containing NETs in the absence or presence of DNase I for additional 12 h. Expression levels of NLRP3 and IL-1β were detected by Western blot. **(A)** One representative result of three independent experiments is shown. **(B)** Relative levels of NLRP3/β-actin **(B)** and IL-1β/β-actin **(C)** were determined by gray value analysis. Data are presented as mean ± SD (n = 3 donors per group). *p < 0.05.

Patients with periodontitis combined with diabetes are prone to develop uncontrolled periodontal inflammation, and then it was investigated whether the increased formation of NETs in high glucose inflammatory environment was involved in the subsequent inflammatory responses in macrophages. To determine whether NETs were able to promote the up-regulation of NLRP3 and IL-1β expressions in macrophages, THP−1-derived macrophages were co-cultured with NET-containing supernatants. The expressions of NLRP3 and IL-1β in macrophages stimulated with NETs from *P. gingivalis* LPS + HG group were significantly increased compared with *P. gingivalis* LPS + NG group (p < 0.05). Moreover, after treatment with DNase I, there were significant decreases in NLRP3 and IL-1β expression levels in THP−1-derived macrophages stimulated with NETs (p < 0.05, **Figure 3**), which suggested that NLRP3 activation in macrophages was attributed to the induction by NETs.

### Store-operated Ca^2+^ entry -mediated Ca^2+^ influx was involved in NET formation in high glucose inflammatory microenvironment

**Figure 4:**
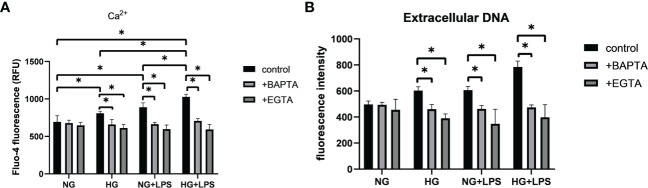
Intracellular Ca^2+^ contributed to the formation of NETs in high glucose inflammatory microenvironment. Neutrophils were pre-incubated with or without Ca^2+^ inhibitors (EGTA or BAPTA), and then stimulated with 1 μg/ml *P. gingivalis* LPS in culture medium with 5.5 mM or 25 mM glucose for 30 min to detect **(A)** intracellular Ca^2+^ levels and 3 h for **(B)** extracellular DNA quantification. Data are presented as mean ± SD (n = 3 donors per group). *p < 0.05.

A common feature of defense responses against invading pathogens in neutrophils is the regulation by Ca^2+^ signaling. SOCE process has been found to initiate Ca^2+^ influx that regulates many biological functions of neutrophils, including the formation of NETs. Stronger fluorescence was observed in HG/*P. gingivalis* LPS group compared with NG group (p < 0.05, **Figure 4A**), indicating the increased intracellular Ca^2+^ levels in the first two groups. Moreover, the level of intracellular Ca^2+^ was remarkably increased in HG + *P. gingivalis* LPS group compared with the rest of the groups (p < 0.05, **Figure 4A**).

To further confirm the effect of Ca^2+^ on NET formation, EGTA and BAPTA were used to chelate free extracellular and intracellular Ca^2+^, respectively. As illustrated in **Figure 4A**, intracellular Ca^2+^ levels were significantly decreased in groups pre-treated with BAPTA or EGTA compared with those without pretreatment (p < 0.05). Moreover, the levels of extracellular DNA in neutrophils pretreated with BAPTA or EGTA were significantly reduced compared with those without Ca^2+^ chelators pretreatment (p < 0.05, **Figure 4B**).

**Figure 5:**
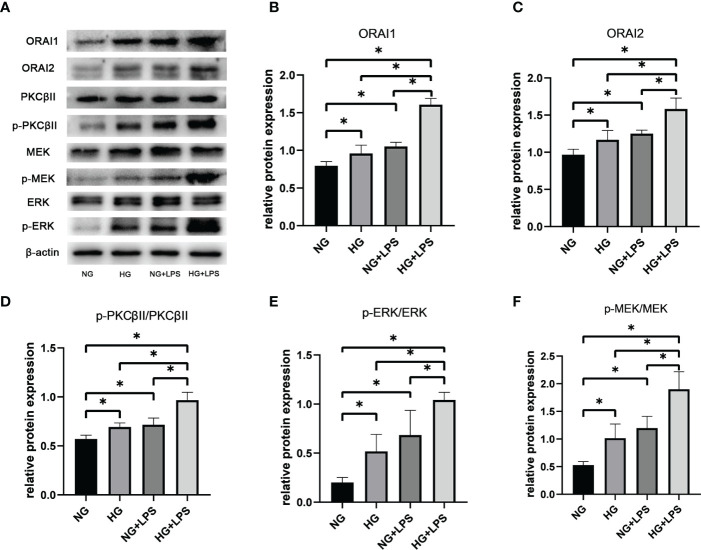
Roles of ORAI1/2, PKC βII, MEK1/2 and ERK1/2 in NET formation induced by *P. gingivalis* LPS in high glucose medium. Neutrophils were stimulated as described in **Figure 1** After 30 min, expression levels of ORAI1, ORAI2, PKC βII, p-PKC βII, MEK1/2, p-MEK1/2, ERK1/2 and p-ERK1/2 were detected by Western blot. **(A)** One representative result of three independent experiments is shown. Relative levels of **(B)** ORAI1/β-actin, **(C)** ORAI2/β-actin, **(D)** p-PKC βII/PKC βII, **(E)** p-MEK1/2/MEK1/2 and **(F)** p-ERK1/2/ERK1/2 were determined by gray value analysis. Data are presented as mean ± SD (n = 3 donors per group). *p < 0.05.

ORAI1 and ORAI2 are the essential components of plasma-membrane calcium-release activated calcium (CRAC) channels in neutrophils, which were controlled by SOCE process. Expressions of ORAI1 and ORAI2 were significantly increased in HG group and *P. gingivalis* LPS group compared with NG group (p < 0.05, **Figures 5A–C**). In addition, protein expression levels of ORAI1 and ORAI2 in HG + *P. gingivalis* LPS group were much higher than both HG group and *P. gingivalis* LPS group (p < 0.05, **Figures 5A–C**).

### Increased phosphorylations of PKC βII, MEK1/2 and ERK1/2 in NET formations in high glucose inflammatory microenvironment

Conventional activation of PKC is Ca^2+^-dependent and activated PKC can be involved in a wide range of cellular activities, including NET formation, *via* MEK1/2 and ERK1/2. Phosphorylation of PKC βII, MEK and ERK1/2 were upregulated in HG group and *P. gingivalis* LPS group compared with the group without any stimulation (p < 0.05, **Figures 5A, D–F**). Moreover, expressions of p-PKC βII, p-MEK and p-ERK1/2 in HG + *P. gingivalis* LPS group were remarkably increased compared with those in HG group and *P. gingivalis* LPS group (p < 0.05, **Figures 5A, D–F**).

### High glucose enhanced *P. gingivalis* LPS-induced NET formation *via* a NADPH oxidase-dependent pathway

**Figure 6:**
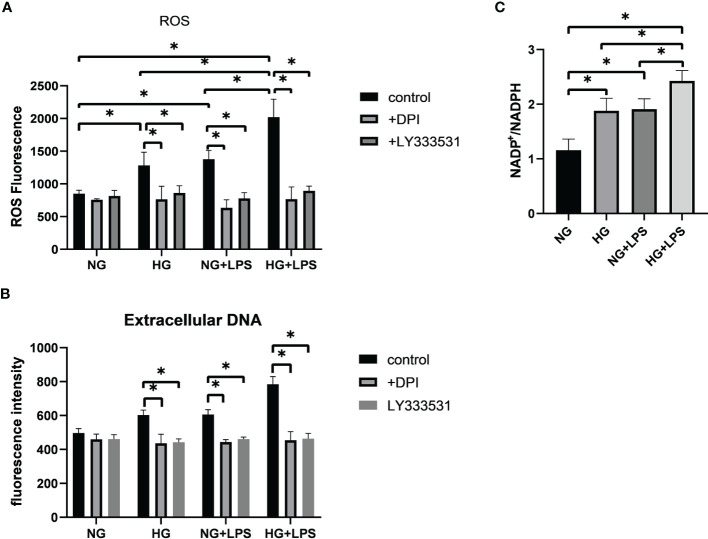
Oxidative stress contributed to the formation of NETs in high glucose inflammatory microenvironment. Neutrophils were pre-treated with 10 μM DPI or 5.9 nM LY333531 for 30 min, and then stimulated with 1 μg/ml *P. gingivalis* LPS in medium containing 5.5 mM or 25 mM glucose. **(A)** ROS production in neutrophils was measured by flow cytometry and **(B)** levels of extracellular DNA were quantified by a microplate reader. **(C)** NADP^+^/NADPH ratio were measured by a microplate reader. Data are presented as mean ± SD (n = 3 donors per group). *p < 0.05.

PKC βII, MEK1/2 and ERK1/2 are involved in NET formation by activating NADPH oxidase (Hakkim et al., 2011). In the above process, ROS was vital by promoting the collapse of nuclear membrane. Thus, a cell permeable fluorescein probe, DCFH-DA, was used to detect the production of ROS. DPI and LY333531 were employed to inhibit NADPH oxidase and PKC βII, respectively. First, HG and *P. gingivalis* LPS stimulations resulted in a greater level of ROS in neutrophils (p < 0.05, **Figure 6A**). The level of ROS in HG + *P. gingivalis* LPS group was remarkably increased compared with the rest of the groups (p < 0.05, **Figure 6A**). Further analysis indicated that the up-regulated levels of ROS and extracellular DNA were significantly suppressed by DPI or LY333531-preincubation (p < 0.05, **Figures 6A, B**).

NADPH oxidase is identified as an important enzyme for respiratory burst in neutrophils. Thus, NADP^+^/NADPH ratio was detected to disclose the activity of NADPH oxidase. The ratio of NADP^+^/NADPH was increased in HG group and *P. gingivalis* LPS group compared with NG group (p < 0.05, **Figure 6C**). Moreover, this ratio was also remarkably increased in HG + *P. gingivalis* LPS group compared with HG group and *P. gingivalis* LPS group (p < 0.05, **Figure 6C**).

## Discussion

In recent years, the influences of the inflammatory microenvironment on NET formation are receiving more and more attentions. Even though the backbone of NETs is similar, there are many differences in the composition and function of NETs depending on distinct stimuli and disorder-specific microenvironment (Mitsios et al., 2016). In our previous study, *P. gingivalis* LPS, one of the classical virulence factors, was demonstrated to trigger the formation of NETs, which was critical in maintaining homeostasis in periodontal tissues (Chen et al., 2022). It has been shown that hyperglycaemic environments *in vitro* and *in vivo* induce the release of NETs (Shafqat et al., 2022). However, NET formation in microenvironment with high glucose and intense inflammation had not been clearly investigated. In this study, enhanced formation of NETs due to high glucose microenvironment combined with *P. gingivalis* LPS-induced inflammation was disclosed for the first time. However, the increased amounts of NETs failed to enhance the bactericidal capacity of neutrophils, but instead amplified inflammatory responses in macrophages.

In a high glucose microenvironment, neutrophils undergo oxidative stress and release a lot of pro-inflammatory cytokines, such as IL-6 and TNF-α, which are strong inducers of NET formation (Dowey et al., 2021; Johnson et al., 2022). Previous studies confirmed that circulating NET components were increased in serum of diabetic patients (Uribe-Querol and Rosales, 2022). Another research also demonstrated that NET formations were exacerbated in high glucose conditions both *in vitro* and *in vivo*, suggesting that high glucose might have an initiating effect on NETs production (Zhu et al., 2021). Additional stimulation, such as lonomycin, further induces a large quantities of NETs in high glucose condition *in vitro*, suggesting that high glucose might have an priming effect on NET formation (Wong et al., 2015). Paradoxically, study of Joshi showed that high glucose induced NET formation, but suppressed further NET production induced by subsequent LPS stimulation (Joshi et al., 2020). The first possible reason for the above-mentioned discrepancy might be the difference in the method of neutrophil isolation. The second possible explanation might be due to the difference in incubation time. The former employed pre-incubation in high glucose culture medium for 1 h, while Joshi selected a 24 h-incubation continuously to mimic the high glucose microenvironment *in vivo*. However, neutrophils are short-lived cells (12-24 h) and should be best stimulated within 2-4 h following collection (Oh et al., 2008; Burn et al., 2021). Pre-incubation for 24 h *in vitro* may have some negative effects on the activity and biological functions of neutrophils. Moreover, the last possible explanation might be the differences in blood glucose levels and complications in diabetic patients whose peripheral venous blood was collected, which may affect the homogeneity of the host. Thus, after neutrophil isolation, stimulation with high glucose and *P. gingivalis* LPS, but without pre-incubation, were employed in this study to avoid any possible effects on neutrophil activities as Menegazzo’s research (Menegazzo et al., 2015).

As the first line of host defense in periodontal tissues, a large number of neutrophils are found in gingival crevice and release NETs, which can trap and kill invading bacteria, fungi and parasites. Jayaprakash’s previous study and ours demonstrated that the bactericidal efficiency of neutrophils against *P. gingivalis* was enhanced by phagocytosis and NETs (Jayaprakash et al., 2015; Chen et al., 2022). However, both bactericidal activity and phagocytic capacity of neutrophils are reduced due to diabetes (Battaglia et al., 2019; Kumar and Dikshit, 2019). Despite the increased formation of NETs in high glucose inflammatory microenvironment, far more *P. gingivalis* survived than those in normal glucose inflammatory group, suggesting that the increased NETs induced by *P. gingivalis* LPS and high glucose did not reverse the impaired bactericidal ability of neutrophils in high glucose microenvironment. Therefore, it might be extremely difficult to control bacterial infection in periodontitis patients combined with diabetes. It should be pointed out that only total bactericidal capacity of neutrophils was assessed in this study, rather than the bactericidal capacity of NETs and there are some other bactericidal strategies, such as phagocytosis, degranulation, respiratory burst and some unknown bactericidal strategies. It is very difficult to evaluate the bactericidal activities of single NETs, excluding the effects of other bactericidal functions.

Macrophages are innate immune cells that are extensively involved in the production of cytokines, as well as the destruction and repair of periodontal tissues. In addition, macrophages recruit and interact with neutrophils in response to invading pathogens, which is necessary for periodontal homeostasis. During the interaction between macrophages and neutrophils, NETs may act as a bridge through inflammasome. Inflammasome is a multi-protein complex activated by various physiological or pathogenic stimuli, such as microorganisms, their virulence factors and danger-associated molecular patterns (DAMPs) (Bullon et al., 2018; Gora et al., 2021). Composition of NLRP3 inflammasome requires NLRP3, apoptosis-associated spec-like protein containing a CARD (ASC), and pro-caspase-1. Active caspase-1 cleaves pro-IL-1β into mature and biologically active IL-1β, which is a potent proinflammatory mediator (Moretti and Blander, 2021). PMA-induced NETs have been shown to upregulate NLRP3 and IL-1β levels *via* a NF-κB signaling pathway in the tumour microenvironment *in vitro* (Wang et al., 2022). In this present study, it was confirmed that NETs further up-regulated NLRP3 and IL-1β expression in a high glucose inflammatory condition. Thus, in addition to impairing bacteria‐killing ability of neutrophils and leading to the spread of infection, excess production of NETs in a high glucose inflammatory microenvironment might further amplify the inflammatory responses and contribute to a persistent immune damage. Therefore, controlling the excessive formation of NETs in periodontitis patients with diabetes may provide a new insight into the management of persistent infections.

Ca^2+^ signals are widely involved in the defense responses of neutrophils against pathogenic microorganisms (Hann et al., 2020). Kenny and Gupta’s studies demonstrated a close link between Ca^2+^ influx and NET induction (Gupta et al., 2014; Kenny et al., 2017). This present study firstly revealed the involvement of Ca^2+^ influx in the formation of NETs in a high glucose inflammatory microenvironment. Ca^2+^ signals are initiated by the SOCE process, which controls the opening of CRAC channels depending on the content of Ca^2+^ stores (Emrich et al., 2022). CRAC channels are composed of ORAI1, ORAI2 and ORAI3, among which ORAI1 and ORAI2 are the major components in neutrophils. Therefore, their deletions impair multiple bactericidal abilities of neutrophils in *S. aureus* skin infection and the formation of NETs against parasite *Neospora caninum* (Zhang et al., 2019; Grimes et al., 2020). In this study, increased protein expressions of ORAI1 and ORAI2 contributed to the enhanced formation of NETs, but not enhanced bactericidal ability, which implied that the components, microstructure and function of NETs remain to be further investigated, as well as whether other bactericidal mechanisms of neutrophils play an important role in high glucose inflammatory microenvironment.

Protein kinase C (PKC) isozymes are members of a Ser/Thr kinase family, which is consists of conventional, novel and atypical isoforms (Kikkawa, 2019). Activation of a conventional PKC isoform, PKC β, is firstly initiated by its phosphorylation, and then binding Ca^2+^ to the C2 domain to recruit itself to the plasma membrane (Newton, 2018). In the development of atherosclerosis, PKC/Raf/MEK/ERK signaling pathway is involved in endothelial cell proliferation triggered by native LDL (N-LDL) (Pintus et al., 2003). PKC β also takes part in the activation of NADPH oxidase and is the main PKC isoforms involved in NET formation (Gray et al., 2013; Damascena et al., 2022). Recent studies indicated that elevated phosphorylation of PKC βII contributed to the enhanced formation of NETs in diabetic patients (Das et al., 2018; Menegazzo et al., 2018). This present study also found that in high glucose inflammatory microenvironment, phosphorylation of PKC βII was enhanced and PKC βII inhibitor, LY333531, further suppressed ROS production and NET formations, confirming the roles of PKC βII in oxidative stress and NET production.

Oxidative stress plays a crucial role in the pathogenesis of both diabetes and periodontitis (Sczepanik et al., 2020; Yaribeygi et al., 2020). ROS is one of the classical signals of NET formation (Vorobjeva and Chernyak, 2020). The major enzyme responsible for ROS production in neutrophils is NADPH oxidase, which is activated by PKCs or MEK/ERK, and then leads to a rapid release of superoxide accompanied by oxidation of NADPH to NADP^+^ (Rabani et al., 2020). Fan’s study demonstrated that NADPH levels were decreased in high glucose-stimulated rat retinal capillary endothelial cells, which resulted in an obvious increase in the NADP^+^/NADPH ratios (Fan et al., 2017). The activation of NADPH oxidase has been demonstrated to participate in NET production, which is triggered by high glucose in patients with diabetic retinopathy (Wang et al., 2019). PKC and Raf-MEK-ERK pathways locate upstream of NADPH oxidase and extensively involved in NET formation (Hakkim et al., 2011). Effects of high glucose and inflammation on ROS production are cumulative, which might be related with the intracellular NADPH oxidase activation and phosphorylation of PKC βII and MEK/ERK. It has been shown that PKC isoforms can phosphorylate the MEK/ERK pathway (Damascena et al., 2022). However, given that there are several isozymes in PKC family and different isozymes do not work in exactly the same way, the relationship between PKC βII and MEK/ERK still needs to be further investigated.

In summary, *P. gingivalis* LPS might contribute to oxidative stress in high glucose microenvironment through a Ca^2+^-PKC-MEK-ERK-NADPH oxidase-ROS pathway, thereby promoting NET formation. Furthermore, enhanced formation of NETs might not reverse the impaired bactericidal activities of neutrophils caused by high glucose, instead lead to a serious inflammatory response in macrophages, which would be a challenge for the control of infection in periodontitis patients with diabetes. Further studies are still needed to explore the roles of high glucose inflammatory microenvironment in NET production *in vivo*, as well as the exact mechanisms involved.

## Data availability statement

The original contributions presented in the study are included in the article/Supplementary Material. Further inquiries can be directed to the corresponding author.

## Ethics statement

The studies involving human participants were reviewed and approved by the Ethical Committee Department, The Affiliated Stomatological Hospital of Nanjing Medical University. The patients/participants provided their written informed consent to participate in this study.

## Author contributions

Conceptualization, YS and YT. Methodology, YT, YX, LF and JS. Validation, YT and YX. Data curation, YT and YX. Writing—original draft preparation, YT. Writing—review and editing, YS. Project administration, YS. Funding acquisition, YS. All authors contributed to the article and approved the submitted version.

## References

[B1] Battaglia M. Petrelli A. Vecchio F. (2019). Neutrophils and type 1 diabetes: current knowledge and suggested future directions. Curr. Opin. Endocrinol. Diabetes Obes. 26, 201–206. doi: 10.1097/MED.0000000000000485 31157631

[B2] Brinkmann V. Reichard U. Goosmann C. Fauler B. Uhlemann Y. Weiss D. S.. (2004). Neutrophil extracellular traps kill bacteria. Science 303, 1532–1535. doi: 10.1126/science.1092385 15001782

[B3] Bullon P. Pavillard L. E. de la Torre-Torres R. (2018). Inflammasome and oral diseases. Exp. Suppl. 108, 153–176. doi: 10.1007/978-3-319-89390-7_7 30536171

[B4] Burn G. L. Foti A. Marsman G. Patel D. F. Zychlinsky A. (2021). The neutrophil. Immunity 54, 1377–1391. doi: 10.1016/j.immuni.2021.06.006 34260886

[B5] Chen J.-L. Tong Y. Zhu Q. Gao L.-Q. Sun Y. (2022). Neutrophil extracellular traps induced by porphyromonas gingivalis lipopolysaccharide modulate inflammatory responses *via* a Ca2+-dependent pathway. Arch. Oral. Biol. 141, 105467. doi: 10.1016/j.archoralbio.2022.105467 35679800

[B6] Chigasaki O. Aoyama N. Sasaki Y. Takeuchi Y. Mizutani K. Ikeda Y.. (2021). Porphyromonas gingivalis, the most influential pathogen in red-complex bacteria: A cross-sectional study on the relationship between bacterial count and clinical periodontal status in Japan. J. Periodontol 92, 1719–1729. doi: 10.1002/JPER.21-0011 33856713

[B7] Damascena H. L. Silveira W. A. A. Castro M. S. Fontes W. (2022). Neutrophil activated by the famous and potent PMA (Phorbol myristate acetate). Cells 11, 2889. doi: 10.3390/cells11182889 36139464 PMC9496763

[B8] Das S. K. Yuan Y.-F. Li M.-Q. (2018). Specific PKC βII inhibitor: one stone two birds in the treatment of diabetic foot ulcers. Biosci. Rep. 38, BSR20171459. doi: 10.1042/BSR20171459 29440456 PMC6127666

[B9] Dowey R. Iqbal A. Heller S. R. Sabroe I. Prince I. R. (2021). A bittersweet response to infection in diabetes; targeting neutrophils to modify inflammation and improve host immunity. Front. Immunol. 12. doi: 10.3389/fimmu.2021.678771 PMC8209466 34149714

[B10] Emrich S. M. Yoast R. E. Trebak M. (2022). Physiological functions of CRAC channels. Annu. Rev. Physiol. 84, 355–379. doi: 10.1146/annurev-physiol-052521-013426 34637326

[B11] Fan C.-L. Qiao Y. Tang M.-K. (2017). Notoginsenoside R1 attenuates high glucose-induced endothelial damage in rat retinal capillary endothelial cells by modulating the intracellular redox state. Drug Des. Devel Ther. 11, 3343–54. doi: 10.2147/DDDT.S149700 PMC5703151 29200830

[B12] Genco R. J. Borgnakke W. S. (2020). Diabetes as a potential risk for periodontitis: association studies. Periodontol 2000 83, 40–45. doi: 10.1111/prd.12270 32385881

[B13] Genco R. J. Graziani F. Hasturk H. (2020). Effects of periodontal disease on glycemic control, complications, and incidence of diabetes mellitus. Periodontol 2000 83, 59–65. doi: 10.1111/prd.12271 32385875

[B14] Giovenzana A. Carnovale D. Phillips B. Petrelli A. Giannoukakis N. (2022). Neutrophils and their role in the aetiopathogenesis of type 1 and type 2 diabetes. Diabetes Metab. Res. Rev. 38, e3483. doi: 10.1002/dmrr.3483 34245096

[B15] Gora I. M. Ciechanowska A. Ladyzynski P. (2021). NLRP3 inflammasome at the interface of inflammation, endothelial dysfunction, and type 2 diabetes. Cells 10, 314. doi: 10.3390/cells10020314 33546399 PMC7913585

[B16] Graves D. T. Ding Z. Yang Y. (2020). The impact of diabetes on periodontal diseases. Periodontol 2000 82, 214–224. doi: 10.1111/prd.12318 31850631

[B17] Gray R. D. Lucas C. D. MacKellar A. Li F. Hiersemenzel K. Haslett C.. (2013). Activation of conventional protein kinase c (PKC) is critical in the generation of human neutrophil extracellular traps. J. Inflammation (Lond) 10, 12. doi: 10.1186/1476-9255-10-12 PMC3643828 23514610

[B18] Grimes D. Johnson R. Pashos M. Cummings C. Kang C. Sampedro G. R.. (2020). ORAI1 and ORAI2 modulate murine neutrophil calcium signaling, cellular activation, and host defense. Proc. Natl. Acad. Sci. U.S.A. 117, 24403–24414. doi: 10.1073/pnas.2008032117 32929002 PMC7533673

[B19] Gupta A. K. Giaglis S. Hasler P. Hahn S. (2014). Efficient neutrophil extracellular trap induction requires mobilization of both intracellular and extracellular calcium pools and is modulated by cyclosporine a. PLoS One 9, e97088. doi: 10.1371/journal.pone.0097088 24819773 PMC4018253

[B20] Hajishengallis G. (2022). Interconnection of periodontal disease and comorbidities: Evidence, mechanisms, and implications. Periodontol 2000 89, 9–18. doi: 10.1111/prd.12430 35244969 PMC9018559

[B21] Hakkim A. Fuchs T. A. Martinez N. E. Hess S. Prinz H. Zychlinsky A.. (2011). Activation of the raf-MEK-ERK pathway is required for neutrophil extracellular trap formation. Nat. Chem. Biol. 7, 75–77. doi: 10.1038/nchembio.496 21170021

[B22] Hann J. Bueb J.-L. Tolle F. Bréchard S. (2020). Calcium signaling and regulation of neutrophil functions: Still a long way to go. J. Leukoc. Biol. 107, 285–297. doi: 10.1002/JLB.3RU0719-241R 31841231

[B23] Herrero-Cervera A. Soehnlein O. Kenne E. (2022). Neutrophils in chronic inflammatory diseases. Cell Mol. Immunol. 19, 177–191. doi: 10.1038/s41423-021-00832-3 35039631 PMC8803838

[B24] Jayaprakash K. Demirel I. Khalaf H. Bengtsson T. (2015). The role of phagocytosis, oxidative burst and neutrophil extracellular traps in the interaction between neutrophils and the periodontal pathogen porphyromonas gingivalis. Mol. Oral. Microbiol. 30, 361–375. doi: 10.1111/omi.12099 25869817

[B25] Johnson J. Jaggers R. M. Gopalkrishna S. Dahdah A. Murphy A. J. Hanssen N. M. J.. (2022). Oxidative stress in neutrophils: implications for diabetic cardiovascular complications. Antioxid Redox Signal 36, 652–666. doi: 10.1089/ars.2021.0116 34148367 PMC9057880

[B26] Joshi M. B. Ahamed R. Hegde M. Nair A. S. Ramachandra L. Satyamoorthy K. (2020). Glucose induces metabolic reprogramming in neutrophils during type 2 diabetes to form constitutive extracellular traps and decreased responsiveness to lipopolysaccharides. Biochim. Biophys. Acta Mol. Basis Dis. 1866, 165940. doi: 10.1016/j.bbadis.2020.165940 32827651

[B27] Kenny E. F. Herzig A. Krüger R. Muth A. Mondal S. Thompson P. R.. (2017). Diverse stimuli engage different neutrophil extracellular trap pathways. Elife 6, e24437. doi: 10.7554/eLife.24437 28574339 PMC5496738

[B28] Kikkawa U. (2019). The story of PKC: A discovery marked by unexpected twists and turns. IUBMB Life 71, 697–705. doi: 10.1002/iub.1963 30393952

[B29] Kumar S. Dikshit M. (2019). Metabolic insight of neutrophils in health and disease. Front. Immunol. 10, 2099. doi: 10.3389/fimmu.2019.02099 31616403 PMC6764236

[B30] Liew P. X. Kubes P. (2019). The neutrophil’s role during health and disease. Physiol. Rev. 99, 1223–1248. doi: 10.1152/physrev.00012.2018 30758246

[B31] Magán-Fernández A. Rasheed Al-Bakri S. M. O’Valle F. Benavides-Reyes C. Abadía-Molina F. Mesa F. (2020). Neutrophil extracellular traps in periodontitis. Cells 9, E1494. doi: 10.3390/cells9061494 PMC7349145 32575367

[B32] Menegazzo L. Ciciliot S. Poncina N. Mazzucato M. Persano M. Bonora B.. (2015). NETosis is induced by high glucose and associated with type 2 diabetes. Acta Diabetol. 52, 497–503. doi: 10.1007/s00592-014-0676-x 25387570

[B33] Menegazzo L. Scattolini V. Cappellari R. Bonora B. M. Albiero M. Bortolozzi M.. (2018). The antidiabetic drug metformin blunts NETosis *in vitro* and reduces circulating NETosis biomarkers *in vivo* . Acta Diabetol. 55, 593–601. doi: 10.1007/s00592-018-1129-8 29546579

[B34] Mitsios A. Arampatzioglou A. Arelaki S. Mitroulis I. Ritis K. (2016). NETopathies? unraveling the dark side of old diseases through neutrophils. Front. Immunol. 7. doi: 10.3389/fimmu.2016.00678 PMC5225098 28123386

[B35] Moretti J. Blander J. M. (2021). Increasing complexity of NLRP3 inflammasome regulation. J. Leukoc. Biol. 109, 561–571. doi: 10.1002/JLB.3MR0520-104RR 32531835 PMC8985609

[B36] Newton A. C. (2018). Protein kinase c: perfectly balanced. Crit. Rev. Biochem. Mol. Biol. 53, 208–230. doi: 10.1080/10409238.2018.1442408 29513138 PMC5901981

[B37] Njeim R. Azar W. S. Fares A. H. Azar S. T. Kfoury Kassouf H. Eid A. A. (2020). NETosis contributes to the pathogenesis of diabetes and its complications. J. Mol. Endocrinol. 65, R65–R76. doi: 10.1530/JME-20-0128 33048064

[B38] Oh H. Siano B. Diamond S. (2008). Neutrophil isolation protocol. J. Vis. Exp. 745. doi: 10.3791/745 PMC3074468 19066523

[B39] Pintus G. Tadolini B. Posadino A. M. Sanna B. Debidda M. Carru C.. (2003). PKC/Raf/MEK/ERK signaling pathway modulates native-LDL-induced E2F-1 gene expression and endothelial cell proliferation. Cardiovasc. Res. 59, 934–944. doi: 10.1016/s0008-6363(03)00526-1 14553833

[B40] Polak D. Sanui T. Nishimura F. Shapira L. (2020). Diabetes as a risk factor for periodontal disease-plausible mechanisms. Periodontol 2000 83, 46–58. doi: 10.1111/prd.12298 32385872

[B41] Rabani R. Cossette C. Graham F. Powell W. S. (2020). Protein kinase c activates NAD kinase in human neutrophils. Free Radic. Biol. Med. 161, 50–59. doi: 10.1016/j.freeradbiomed.2020.09.022 33011272

[B42] Ravindran M. Khan M. A. Palaniyar N. (2019). Neutrophil extracellular trap formation: physiology, pathology, and pharmacology. Biomolecules 9, E365. doi: 10.3390/biom9080365 PMC6722781 31416173

[B43] Sabbatini M. Magnelli V. Renò F. (2021). NETosis in wound healing: When enough is enough. Cells 10, 494. doi: 10.3390/cells10030494 33668924 PMC7996535

[B44] Salhi L. Reners M. (2022). Update on the bidirectional link between diabetes and periodontitis. Adv. Exp. Med. Biol. 1373, 231–240. doi: 10.1007/978-3-030-96881-6_12 35612801

[B45] Sczepanik F. S. C. Grossi M. L. Casati M. Goldberg M. Glogauer M. Fine N.. (2020). Periodontitis is an inflammatory disease of oxidative stress: We should treat it that way. Periodontol 2000 84, 45–68. doi: 10.1111/prd.12342 32844417

[B46] Shafqat A. Abdul Rab S. Ammar O. Al Salameh S. Alkhudairi A. Kashir J.. (2022). Emerging role of neutrophil extracellular traps in the complications of diabetes mellitus. Front. Med. (Lausanne) 9. doi: 10.3389/fmed.2022.995993 PMC9445264 36082273

[B47] Silvestre-Roig C. Fridlender Z. G. Glogauer M. Scapini P. (2019). Neutrophil diversity in health and disease. Trends Immunol. 40, 565–583. doi: 10.1016/j.it.2019.04.012 31160207 PMC7185435

[B48] Tsai C.-Y. Hsieh S.-C. Liu C.-W. Lu C.-S. Wu C.-H. Liao H.-T.. (2021). Cross-talk among polymorphonuclear neutrophils, immune, and non-immune cells *via* released cytokines, granule proteins, microvesicles, and neutrophil extracellular trap formation: A novel concept of biology and pathobiology for neutrophils. Int. J. Mol. Sci. 22, 3119. doi: 10.3390/ijms22063119 33803773 PMC8003289

[B49] Uribe-Querol E. Rosales C. (2022). Neutrophils actively contribute to obesity-associated inflammation and pathological complications. Cells 11, 1883. doi: 10.3390/cells11121883 35741012 PMC9221045

[B50] Vitkov L. Minnich B. Knopf J. Schauer C. Hannig M. Herrmann M. (2020). NETs are double-edged swords with the potential to aggravate or resolve periodontal inflammation. Cells 9, 2614. doi: 10.3390/cells9122614 33291407 PMC7762037

[B51] Vorobjeva N. V. Chernyak B. V. (2020). NETosis: molecular mechanisms, role in physiology and pathology. Biochem. (Mosc) 85, 1178–1190. doi: 10.1134/S0006297920100065 PMC7590568 33202203

[B52] Wang Y. Liu F. Chen L. Fang C. Li S. Yuan S.. (2022). Neutrophil extracellular traps (NETs) promote non-small cell lung cancer metastasis by suppressing lncRNA MIR503HG to activate the NF-κB/NLRP3 inflammasome pathway. Front. Immunol. 13, 867516. doi: 10.1038/nm.3887 35707534 PMC9190762

[B53] Wang J. Zhou Y. Ren B. Zou L. He B. Li M. (2021). The role of neutrophil extracellular traps in periodontitis. Front. Cell Infect. Microbiol. 11. doi: 10.3389/fcimb.2021.639144 PMC8012762 33816343

[B54] Wang L. Zhou X. Yin Y. Mai Y. Wang D. Zhang X. (2019). Hyperglycemia induces neutrophil extracellular traps formation through an NADPH oxidase-dependent pathway in diabetic retinopathy. Front. Immunol. 9, 3076. doi: 10.3389/fimmu.2018.03076 30671057 PMC6331470

[B55] Wong S. L. Demers M. Martinod K. Gallant M. Wang Y. Goldfine A. B. (2015). Diabetes primes neutrophils to undergo NETosis, which impairs wound healing. Nat Med. 21, 815–19. doi: 10.1038/nm.3887 PMC4631120 26076037

[B56] Xu W. Zhou W. Wang H. Liang S. (2020). Roles of porphyromonas gingivalis and its virulence factors in periodontitis. Adv. Protein Chem. Struct. Biol. 120, 45–84. doi: 10.1016/bs.apcsb.2019.12.001 32085888 PMC8204362

[B57] Yaribeygi H. Sathyapalan T. Atkin S. L. Sahebkar A. (2020). Molecular mechanisms linking oxidative stress and diabetes mellitus. Oxid. Med. Cell Longev 2020, 8609213. doi: 10.1155/2020/8609213 32215179 PMC7085395

[B58] Zhang B. Guo H. Yang W. Li M. Zou Y. Loor J. J.. (2019). Effects of ORAI calcium release-activated calcium modulator 1 (ORAI1) on neutrophil activity in dairy cows with subclinical hypocalcemia1. J. Anim. Sci. 97, 3326–3336. doi: 10.1093/jas/skz209 31299068 PMC6667259

[B59] Zhu S. Yu Y. Ren Y. Xu L. Wang H. Ling X.. (2021). The emerging roles of neutrophil extracellular traps in wound healing. Cell Death Dis. 12, 984. doi: 10.1038/s41419-021-04294-3 34686654 PMC8536667

